# Self-assembled monolayer of designed and synthesized triazinedithiolsilane molecule as interfacial adhesion enhancer for integrated circuit

**DOI:** 10.1186/1556-276X-6-483

**Published:** 2011-08-03

**Authors:** Fang Wang, Yanni Li, Yabin Wang, Zhuo Cao

**Affiliations:** 1 College of Science, Northwest Agriculture and Forest University, Xi Nong Road No. 22, Yangling, Shaanxi 712100, China

**Keywords:** adhesion, copper, diffusion barrier, self-assembled monolayer, surface chemistry

## Abstract

Self-assembled monolayer (SAM) with tunable surface chemistry and smooth surface provides an approach to adhesion improvement and suppressing deleterious chemical interactions. Here, we demonstrate the SAM comprising of designed and synthesized 6-(3-triethoxysilylpropyl)amino-1,3,5-triazine-2,4-dithiol molecule, which can enhance interfacial adhesion to inhibit copper diffusion used in device metallization. The formation of the triazinedithiolsilane SAM is confirmed by X-ray photoelectron spectroscopy. The adhesion strength between SAM-coated substrate and electroless deposition copper film was up to 13.8 MPa. The design strategy of triazinedithiolsilane molecule is expected to open up the possibilities for replacing traditional organosilane to be applied in microelectronic industry.

## Introduction

Isolating individual components of nanoscale architectures comprised of thin films or nanostructures is a critical challenge in micro- and nanoscale device fabrication [1]. One important example that illustrates this challenge could be seen in Cu-interconnected sub-100-nm device structures, which require less than 5-nm-thick interfacial layers to inhibit Cu diffusion into adjacent dielectrics [2]. Conventional interfacial barrier layers such as TaN, Ta, Ti, TiN, or W have already been optimized in microelectronic applications. However, such "thick" layers are not suitable for micro- and nanoscale device fabrication, and the above materials cannot form uniform and continuous film below 5 nm in thickness [3]. The barrier layer thickness must be minimized while maintaining high-performance diffusion barrier properties and good adhesion strength with neighboring layers [4]. Another significant example is seen in the adhesion between copper and substrate in printed circuit board technology.

An alternative to the above interfacial layer is the organic self-assembled monolayer (SAM) [5] with sub-nanometer dimensions. The SAM [6,7] composed of short aliphatic chains with desired terminal function groups has been investigated by modifying surface properties for the above requirement. The selectivity and adhesion strength between the function group of SAM and the substrate impact the film packing density and thermal stability, and the chain length also has influence on the packing density and order. In recent years, G. Ramanath has reported the technique of fabricating SAM with the organosilanes as Cu diffusion barrier layer, and interfacial adhesion in microelectronics devices [2,8-12]. The results showed that the SAM inhibited Cu diffusion into substrate interface and enhanced the interfacial adhesion to increase the device lifetime [8,13]. This technique has two advantages: (a) a strong interfacial bonding which can immobilize Cu, and (b) the creation of a vacuum-like potential barrier between Cu and the dielectric layer to inhibit Cu ionization and transport [14]. The former can be achieved through strong, local chemical interaction by choosing appropriate terminal groups, and the latter can be accomplished by using SAM with suitable chain length or the introduction of aromatic group. This technique offers the potential for tailoring effective barriers with decreased thickness.

The organosilane molecules used to fabricate SAM as functional interfacial layer have been widely investigated. Table 1 shows chemical formula and nomenclature of the self-assembled organosilanes commonly used in electronic industry or previous researches.

**Table 1:** Chemical formula and nomenclature of the self-assembled organosilanes usually used in previous researches

Molecule	Chemical formula	Name
SAM1	HSCH_2_CH_2_CH_2_Si(OCH_3_)_3_	(3-Mecaptopropyl)trimethoxysilane
SAM2^12^	H_2_NCH_2_CH_2_CH_2_Si(OCH_3_)_3_	(3-Aminopropyl)trimethoxysilane
SAM3	CH_3_CH_2_CH_2_Si(OCH_3_)_3_	(*n*-Propyl)trimethoxysilane
SAM4		3-[2-(Trimethoxysilyl)ethyl]pyridine
SAM5		2-(Trimethoxysilyl)ethylbenzene
SAM6		Phenyltrimethoxysilane
SAM7^13^		2-(Diphenylphosphino)ethyltriethoxysilane

From the results of Table 1, we can learn that the mercapto-silane (SAM1), amino-silane (SAM2 [15]), and pyridyl-silane (SAM4) are excellent coupling agents between substrates and Cu to act as interfacial adhesion enhancer. Compared with aliphatic groups, larger volume organosilanes with the aromatic ring (SAM4, SAM5, SAM6, and SAM7 [16]) sterically hinder copper diffusion. Therefore, the ideal organosilane molecules should be with both aromatic ring and terminal function group which have high reactivity with copper. In recent years, studies have been mainly focused on the approaches of Cu metallization including chemical vapor deposition, physical vapor deposition, electroless deposition (ELD), and electroplating. Cu ELD was especially emphasized in future interconnect technology. However, the organosilanes utilized in this technique were mainly mercaptopropyltrimethoxysilane (MPTMS; SAM1 [3,9-11]) and aminopropyltrimethoxysilane (APTMS; SAM2 [4,9,17]). So far, no research has been carried out on the modification of organosilane, and it is necessary to design and synthesize functional organosilane molecule.

**Figure 1:**
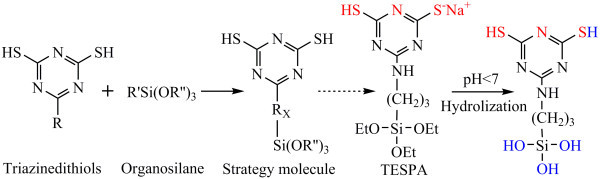
**A strategy schema and designed molecule**. (R = Cl; R' = NH_2_(CH_2_)_3_; R" = CH_2_CH_3_; Rx = NH (CH_2_)_3_).

Our group has focused on the triazinedithiols (TDTs) [18-20] for many years. With two mercapto groups and aromatic ring with nitrogen atom, TDTs combine the advantages of SAM1 and SAM2, which have high reactivity with copper. Besides, nitrogen atom that existed between the two mercapto groups is different with SAM4, which possesses a better space position for copper immobilization. But the triazinedithiols could not react with the substrate for lack of silane group (Si-(OR)_3_). Therefore, our research concentrates on the combination of triazinedithiols and silane (see Figure 1).

In this paper, we demonstrate a designed and synthesized triazinedithiolsilane molecule - 6-(3-triethoxysilylpropyl)amino-1,3,5-triazine-2,4-dithiol monosodium salt (abbreviation, TESPA, see Figure 1) according to the strategies mentioned. TESPA has three active sites which refer to two mercapto groups [21] and a nitrogen atom. We also preliminarily investigate the adhesion strength between ELD copper film and TESPA SAM to verify whether it can be used as adhesion enhancer and diffusion barrier in device applications.

## Experimental

The chemical structure of TESPA was identified by nuclear magnetic resonance (NMR), Fourier transform infrared spectroscopy (FT-IR), and mass spectroscopy (MS) (The data are available in Additional file 1). ^1^H NMR and ^13^C NMR spectra were recorded by Bruker AC 400 with 500 MHz (Bruker Daltonics, Billerica, MA, USA). FT-IR spectra were measured using Bruker TENSOR 37 (Bruker Daltonics). MS was recorded by LCQ-Fleet (Thermo Scientific, Waltham, MA, USA).

TESPA SAM was fabricated on epoxy resin surface. The epoxy resin surface was treated by corona discharge for 10 s to facilitate the formation of SAM through surface hydroxylation. TESPA SAM was obtained by dipping the epoxy resin into 2.5 mM TESPA monomer ethanol-water (*V*/*V *= 95:5) solution for 5 min at room temperature. The substrate was dried with nitrogen gas and cured at 120°C for 20 min. Then, a Sn-Pd^II ^colloidal solution was used as a catalyst precursor [22], which was prepared via precise control of sequential hydrolysis of Pd^II ^species according to the hydrolysis mechanism of Pd^II ^salts in a chloride-rich aqueous solution. The electroless deposition bath was prepared according to recent study [23]. X-ray photoelectron spectroscopy (XPS) was performed to investigate the elemental composition of surface by using ULVAC PHI-5600 spectrometer (Ulvac Technologies, Inc., Methuen, MA, USA). The adhesion strength between ELD copper film and TESPA SAM-coated epoxy resin substrate was investigated by T-peel test using an autograph S-100 apparatus (Shimadzu Corporation, Kyoto, Japan).

## Results and discussion

TESPA was synthesized by the reaction of cyanuric chloride, 3-aminopropyltriethoxy silane (APTES), and NaSH according to the strategy described in the Figure 1. The stirring tetrahydrofuran (THF) solution of cyanuric chloride (0.1 mol) was added with APTES (0.1 mol) over a period of 60 min. And then the reaction mixture was added with THF solution of triethylamine (0.12 mol) for 1-day reaction. After, the solvent was removed under vacuum to yield 6-(3-triethoxysilylpropyl)amino-1,3,5-triazine-2,4-dichloride. NaSH ethanol solution was added dropwise for 2-h reaction to the ethanol solution of the dichloride. After, the solvent was removed under vacuum to yield TESPA. Yield was 75.6%, and m.p. > 203°C. Elemental analysis calculation for C_12_H_23_N_4_S_2_O_3_NaSi was: C, 37.29%; H, 6.00%; N, 14.49%; however, found was: C, 37.46%; H, 6.03%; and N, 14.44%. The results of NMR, FT-IR, and MS also suggest that TESPA have been synthesized (see Additional file 1).

**Figure 2:**
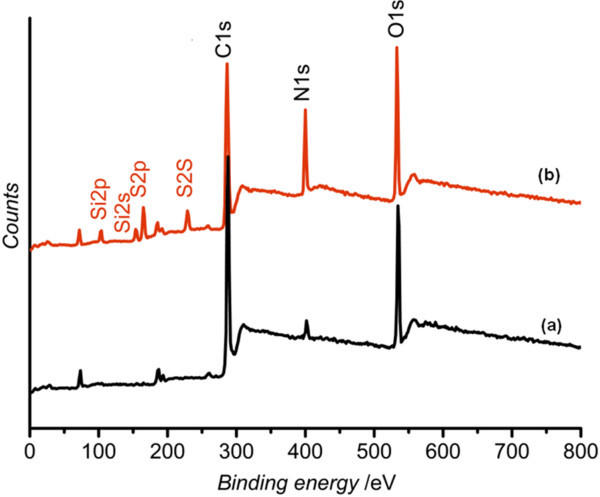
**XPS survey spectra of epoxy resin surface: (a) uncoated and (b) TESPA SAM-coated**. X-ray source is monochromated Al Kα ray. Testing area is 800 × 2,000 μm. Takeoff angle is 45°. The pressure in the preparation chamber is less than 10^-7 ^Torr and less than 4 × 10^-10 ^Torr in the analysis chamber.

The XPS spectra of untreated and TESPA-treated epoxy resin substrate are shown in Figure 2. It can be seen that only the peaks of C1s, O1s, and N1s are observed for the untreated substrate, while the peaks of N1s, S2s, S2p, Si2s, and Si2p corresponding to the TESPA SAM-covered substrate. The results confirmed the formation of the TESPA SAM on the epoxy resin substrate. It can be concluded that the Si-OH groups of hydrolyzed TESPA (see Figure 1) react with the polar groups on the pre-treated epoxy resin surface to form the TESPA SAM. The thickness of the TESPA SAM was about 2.8 nm.

**Figure 3:**
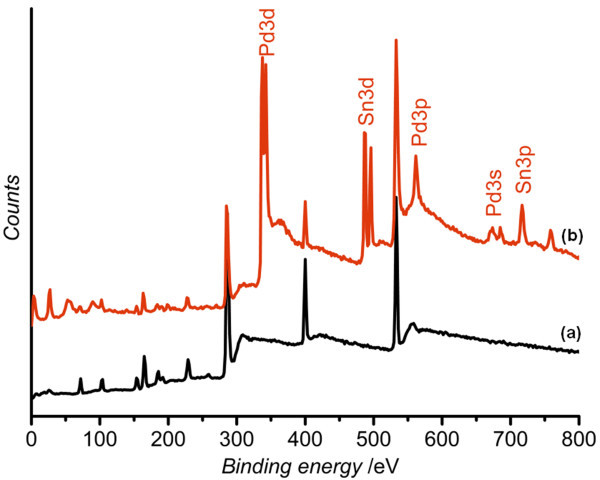
**XPS survey spectra of TESPA SAM-coated epoxy resin surface**. (a) Before Pd catalyzation and (b) after Pd catalyzation.

The results of XPS for the TESPA SAM before and after Pd catalyzation are shown in Figure 3. The presence of Sn3p, Sn3d, Pd3s, Pd3p, and Pd3d peaks suggests the adsorption of catalyst to TESPA SAM-coated surface, and the designed TESPA molecule covalently binds colloidal Pd^II ^catalysts, which can promote ELD copper film onto the TESPA SAM-coated surface [22].

**Figure 4:**
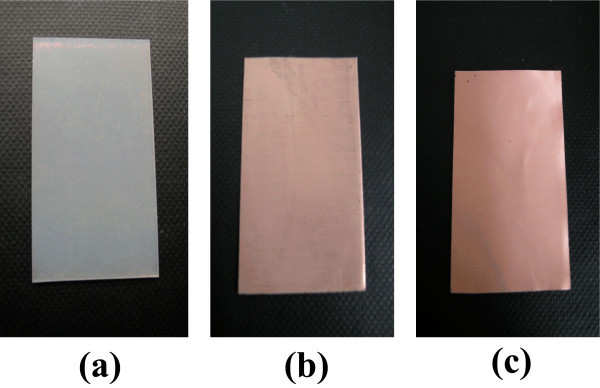
**Surface images of epoxy resin**. (a) The uncoated surface without ELD copper film, (b) ELD copper film on the uncoated surface, and (c) ELD copper film on the TESPA SAM uncoated surface.

The surface image of TESPA SAM-coated epoxy resin substrate after ELD copper is shown in Figure 4. It can be seen that the surface is uniform and compact. The adhesion strength between TESPA SAM-coated epoxy resin and ELD copper film was up to 13.8 MPa, which could satisfy the purpose of TESPA SAM as adhesion enhancer and diffusion barrier layer, while the adhesion strength between non-TESPA-treated substrate and ELD copper film was only 1.2 MPa. It is clearly indicated that the TESPA SAM can be applied as interfacial adhesion enhancer and diffusion barrier. It is expected that TESPA will probably replace the traditional organosilane (MPTMS, APTMS, etc) to be applied in microelectronic industry. However, the interaction mechanism of two mercapto groups and nitrogen atoms in TESPA with copper remains to be studied. Also, the test [23] of leakage current density (*j*_leakage_) as a function of time during bias thermal annealing (BTA, *t*_BTA_) will be carried out. In order to understand the Cu-TESPA interface chemistry, XPS on Cu/TESPA/SiO_2_/Si structure will also be studied in the future research.

## Conclusion

The functional triazinedithiolsilane molecule TESPA was designed and synthesized. The Si-OH group of hydrolyzed TESPA could react with the polar groups on pretreated epoxy resin surface to form the TESPA SAM, which promote ELD copper film onto the substrate. The adhesion strength between TESPA SAM-coated epoxy resin and ELD copper film was up to 13.8 MPa, which could satisfy the purpose of TESPA SAM applied as adhesion enhancer. The design strategy of TESPA will provide possibilities for replacing the traditional organosilane (MPTMS, APTMS, etc.) to be applied in microelectronic industry.

## Competing interests

The authors declare that they have no competing interests.

## Authors' contributions

FW designed the experimental idea and synthetic strategy. YL and ZC participated in the synthesis and characterization of the target molecule, and performed the statistical analysis. YW participated in the design of the study and drafted the manuscript. All authors read and approved the final manuscript.

## Supplementary Material

Additional file 1 **Spectral data of TESPA**. The spectral data of FT-IR, ^1^H NMR and ^13^C NMR and MS for TESPA. Click here for file

## References

[B1] Kuech TF Mawst LJ Nanofabrication of III-V semiconductors employing diblock copolymer lithography J Phys D: Appl Phys 2010 43 183001 10.1088/0022-3727/43/18/183001

[B2] Gandhi D Lane M Zhou Y Singh A Nayak S Tisch U Eizenberg M Ramanath G Annealing-induced interfacial toughening using a molecular nanolayer Nature 2007 447 299 10.1038/nature05826 17507979

[B3] Doppelt P Semaltianos N Deville Cavellin C Pastol J Ballutaud D High affinity self-assembled monolayers for copper CVD Microelectron Eng 2004 76 113 10.1016/j.mee.2004.07.023

[B4] Caro A Armini S Richard O Maes G Borghs G Whelan C Travaly Y Bottom-up engineering of subnanometer copper diffusion barriers using NH_2_-derived self-assembled monolayers Adv Funct Mater 2010 20 1125 10.1002/adfm.200902072

[B5] Liu G Zhao H Zhang J Park JH Mawst LJ Tansu N Selective area epitaxy of ultra-high density InGaN quantum dots by diblock copolymer Nanoscale Res Lett 2011 6 342 10.1186/1556-276X-6-342 21711862 PMC3211431

[B6] Moores B Simons J Xu S Leonenko Z AFM-assisted fabrication of thiol SAM pattern with alternating quantified surface potential Nanoscale Res Lett 2011 6 185 10.1186/1556-276X-6-185 21711703 PMC3211238

[B7] Demirel G Caglayan MO Garipcan B Duman M Piskin E Formation and organization of amino terminated self-assembled layers on Si(001) surface Nanoscale Res Lett 2007 2 350 10.1007/s11671-007-9071-7

[B8] Krishnamoorthy A Chanda K Murarka S Ramanath G Ryan J Self-assembled near-zero-thickness molecular layers as diffusion barriers for Cu metallization Appl Phys Lett 2001 78 2467 10.1063/1.1365418

[B9] Gandhi D Ganesan P Chandrasekar V Gan Z Mhaisalkar S Li H Ramanath G Molecular-nanolayer-induced suppression of in-plane Cu transport at Cu-silica interfaces Appl Phys Lett 2007 90 163507 10.1063/1.2722667

[B10] Gandhi D Tisch U Singh B Eizenberg M Ramanath G Ultraviolet-oxidized mercaptan-terminated organosilane nanolayers as diffusion barriers at Cu-silica interfaces Appl Phys Lett 2007 91 143503 10.1063/1.2760164

[B11] Ganesan P Kumar A Ramanath G Surface oxide reduction and bilayer molecular assembly of a thiol-terminated organosilane on Cu Appl Phys Lett 2005 87 011905 10.1063/1.1968414

[B12] Garg S Singh B Teki R Lane M Ramanath G Hydrophobic fluoroalkylsilane nanolayers for inhibiting copper diffusion into silica Appl Phys Lett 2010 96 143121 10.1063/1.3374453

[B13] Hu M Noda S Tsuji Y Okubo T Yamaguchi Y Komiyama H Effect of interfacial interactions on the initial growth of Cu on clean SiO2 and 3-mercaptopropyltrimethoxysilane-modified SiO2 substrates J Vac Sci Technol A 2002 20 589 10.1116/1.1458941

[B14] McBrayer J Swanson R Sigmon T Diffusion of metals in silicon dioxide J Electrochem Soc 1986 133 1242 10.1149/1.2108827

[B15] Ganesan P Singh A Ramanath G Diffusion barrier properties of carboxyl-and amine-terminated molecular nanolayers Appl Phys Lett 2004 85 579 10.1063/1.1775035

[B16] Mikami N Hata N Kikkawa T Machida H Robust self-assembled monolayer as diffusion barrier for copper metallization Appl Phys Lett 2003 83 5181 10.1063/1.1635665

[B17] Rebiscoul D Perrut V Morel T Jayet C Cubitt R Haumesser P Alkoxysilane layers compatible with copper deposition for advanced semiconductor device applications Langmuir 2010 26 8981 10.1021/la904771s 20187647

[B18] Wang F Wang Y Li Y Wang Q Preparation of triazinedithiol polymeric nanofilm by two-step potentiostatic polymerization technique on aluminum surface Mater Lett 2010 65 621

[B19] Wang F Mori K Kang Z Oishi Y Magnetic field effects on the polymerization of 6-*N, N*-dioctylamino-1,3,5-triazine-2,4-dithiol Heteroat Chem 2007 18 60 10.1002/hc.20257

[B20] Mori K Suzuki K Shimizu K Oishi Y Evaporation polymerization of 6-dibutylamino-1,3,5-triazine-2,4-dithiol on iron plates Langmuir 2002 18 9527 10.1021/la020301h

[B21] Rezaee A Pavelka LC Mittler S Binary mixtures of SH- and CH3-terminated self-assembled monolayers to control the average spacing between aligned gold nanoparticles Nanoscale Res Lett 2009 4 1319 10.1007/s11671-009-9399-2 20628461 PMC2894174

[B22] Brandow S Chen M Aggarwal R Dulcey C Calvert J Dressick W Fabrication of patterned amine reactivity templates using 4-chloromethylphenylsiloxane self-assembled monolayer films Langmuir 1999 15 5429 10.1021/la9902082

[B23] Fujiwara Y Kobayashi Y Sugaya T Koishikawa A Hoshiyama Y Miyake H Adsorption promotion of Ag nanoparticle using cationic surfactants and polyelectrolytes for electroless Cu plating catalysts J Electrochem Soc 2010 157 D211 10.1149/1.3306025

