# Supplementary material for: Self-assembled monolayer of designed and synthesized triazinedithiolsilane molecule as interfacial adhesion enhancer for integrated circuit
Source: Nanoscale Res Lett. 2011 Aug 3;6(1):483. doi: 10.1186/1556-276X-6-483 (PMC3211997; doi:10.1186/1556-276X-6-483)
Supplement: Additional file 1 — Spectral data of TESPA. The spectral data of FT-IR, 1H NMR and 13C NMR and MS for TESPA. [file 1556-276X-6-483-S1.DOC]

***Supporting information***

**Self-assembled Monolayer of Designed and Synthesized Triazinedithiolsilane Molecule as Interfacial Adhesion Enhancer for Integrated Circuit**

*Fang Wang***, Yanni Li, Yabin Wang, Zhuo Cao*

College of science, Northwest Agriculture & Forest University, Xi Nong Road No.22, Yangling, Shaanxi 712100, China

*To whom correspondence should be addressed. Emails: wangfang4070@nwsuaf.edu.cn; wf4070@yahoo.com.cn

Spectral data of TESPA are shown as follows:

**^1^H-NMR** (500 MHz, DMSO-d*6*, 293K, TMS): δ 0.55 (t, ***J*** = 8.5 Hz, 2H; CH_2_Si), 1.10~1.16 (m, ***J*** = 7.0 Hz, 9H; CH_3_), 1.46~1.52 (m, 2H; CH_2_CH_2_CH_2_), 3.15 ~ 3.17 (m, 2H; CH_2_N), 3.75 (m, ***J*** = 7.0 Hz, 6H; CH_2_O), 7.19 (t, ***J*** = 5.5 Hz, 1H; NH), 10.24 (s, 1H; SH).


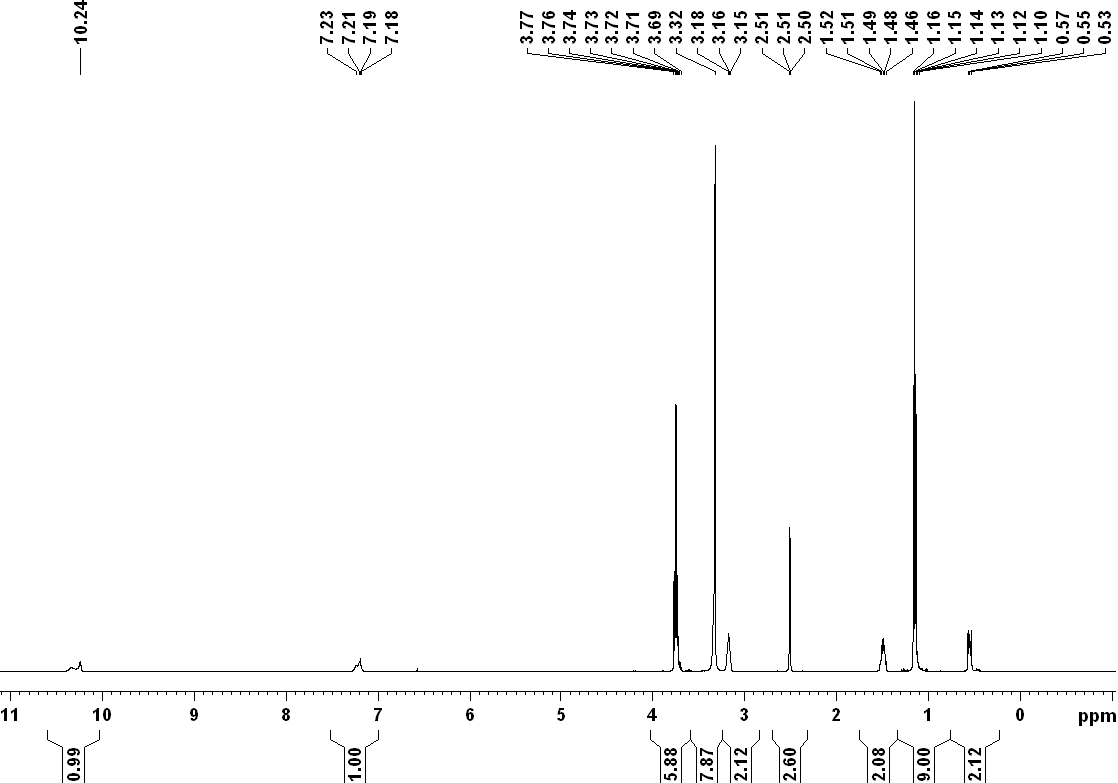


**^13^C-NMR** (500 MHz, DMSO-d*6*, 293K, TMS): δ 7.79, 18.78, 23.24, 43.21, 58.18, 160.79, 181.30, 182.91.


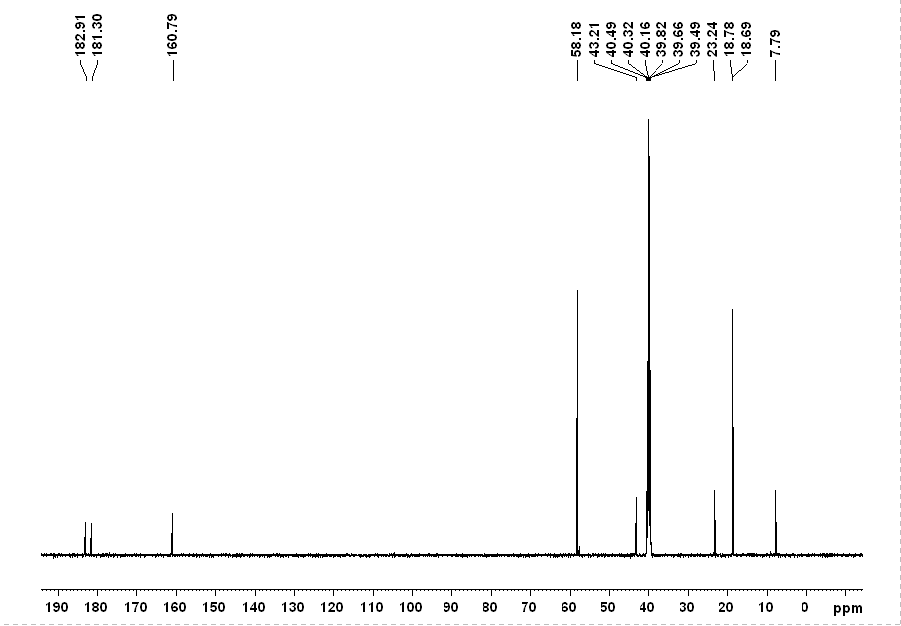


**ESI-MS**: *m/z* (%): 387.06 (100) [*M*^+^+H]

**FT-IR** (KBr): *ν*^～^= 3484, 3227 (w; ν(NH)), 2974 (w), 2930 (w), 1582 (m; ν(C=N)), 1502 (s), 1461 (s), 1376 (m), 1064 (m; ν(SiOCH_3_)), 770 (m; ν(SiCH_2_)), 657 cm^-1^ (w; ν(SH)).
